# The experiences of culturally and linguistically diverse health practitioners in dominant culture practice: a scoping review

**DOI:** 10.1007/s10459-024-10359-7

**Published:** 2024-07-22

**Authors:** Mikaela Harris, Timothea Lau-Bogaardt, Fathimath Shifaza, Stacie Attrill

**Affiliations:** 1 College of Nursing and Health Sciences, Flinders University, Level 1, Room N103, Sturt North Sturt Rd, Bedford Park, SA 5042 Australia; 2 School of Allied Health Science and Practice, The University of Adelaide, Level 4, Engineering Maths and Science Building North Terrace Campus, Adelaide, SA 5005 Australia

**Keywords:** Discrimination, Health professions, Practice community, Legitimate peripheral participation, Community of practice

## Abstract

Increasing the proportion of culturally and linguistically diverse (CALD) health practitioners is identified as one strategy to address healthcare disparities that individuals from minority or under-represented backgrounds experience. However, professional and institutional cultures and structures are known to contribute to the challenges for CALD practitioners who work in dominant culture practice contexts. This scoping review used the theory of Legitimate Peripheral Participation to describe and interpret literature about the experiences of CALD health practitioners in view of informing strategies to increase their representation. A systematised search was conducted across four allied health, medicine and nursing databases. Following abstract and full text screening, articles which fit the inclusion criteria (n = 124) proceeded to data extraction. Categories relating to the experiences of practitioners were extracted, and three themes were identified that were subsequently theoretically interpreted: Discrimination, Consequences and Hierarchy. Discrimination functioned as a barrier to CALD practitioners being legitimised and able to participate equally in healthcare practice, retaining their position at the periphery of the practice community; Consequences reinforced this peripheral position and further impeded legitimation and participation; and Hierarchy was maintained through structures that reinforced and reproduced these barriers. The findings summarise how these barriers are reinforced through the intersections of professional and racial hierarchies, and highlight a need for strategies to address discrimination and structures that marginalise CALD practitioners’ identity, practices and participation in their health professional communities.

**Supplementary Information:**

The online version contains supplementary material available at 10.1007/s10459-024-10359-7.

## Background

The professionalisation of medicine, nursing and allied health has contributed to improving patient safety, increasing accountability, and expanding knowledge (Gunderman & Streicher, 2012; Khalili et al., 2014). However, the resultant professional cultures may also perpetuate pre-existing inequities, as knowledge generated by professionalisation derives from the gender, class and racial hierarchies of the context in which they develop (Grosfoguel, 2002; Khalili et al., 2014). Understanding how practitioners from under-represented backgrounds operate within the dominant culture contexts of their health professions assists to shine light on both the nature and impact of this; and enables the unpacking of the structures that sustain these hierarchies from the perspectives of the individuals most affected by them. This review critically examines the literature describing experiences of health practitioners from culturally and/or linguistically diverse (CALD) backgrounds and their experiences in dominant culture healthcare settings. In this paper, health professionals who are culturally and linguistically diverse include those from populations who are not from the dominant cultural and/or linguistic population of the country in which they practice or work. This may include individuals from racial/ethnic/cultural backgrounds that differ to the dominant group within a country, and/or those who speak a home language that differs from the official language of the country, or of health practice. This definition is intended to capture how individuals who are not from the dominant culture/ethnicity/race/language of a country experience healthcare practice, and is not limited to practice in white, English-speaking contexts.

In Western countries, a lack of diversity, not just in the health workforce, but in organisational and leadership roles has led to a system that is designed by and for the dominant white population, and disadvantages minority groups (Abrahams et al., 2019; Betancourt et al., 2003). Whilst many professional bodies have prioritised strategies to recruit and retain CALD practitioners (e.g. American Association of Colleges of Nursing, 2019; Australian Physiotherapy Association, 2020; Canadian Medical Association, 2021; Royal College of General Practitioners, 2021), to date, actions have failed to ensure health professions represent the communities they serve (Colby & Ortman, 2015). Existing research suggests that the experiences of CALD practitioners are underreported and misunderstood (Wyatt et al., 2021), and limited evidence that CALD health workforce participation has substantially increased suggests that current strategies to problematise and draw solutions may be failing to address deeply recursive social structures (Kyere & Fukui, 2023). Health workforce diversity has been addressed in recent review studies that have mapped workforce diversity (e.g. physician workforce Silver et al., 2019); surgical workforce (Burks et al., 2022); and broader health workforce, including nursing and allied health (Wilbur, et al., 2020), and in studies that have critically examined racialized organisational structures that impede workforce diversity (Kyere & Fukui, 2023). These review studies have examined the implications of continued poor workforce diversity on healthcare outcomes for CALD minority populations, but have not explored the CALD health practitioner’s experiences in practice within dominant culture healthcare settings. A synthesis of this latter literature may illuminate how, where and why structures and hierarchies of dominant cultures in healthcare are most impactful, and how CALD individuals respond and adapt to accommodate their experiences. These insights are critical, and largely missing voices in problematising why health workforce diversity remains stubbornly unaddressed, and to inform education, interventions and culture change strategies.

In this scoping review study, literature describing the experiences of CALD practitioners in dominant practice contexts is interpreted using the theory of Legitimate Peripheral Participation (LPP) (Lave & Wenger, 1991). This theory posits that learning is an outcome of participating in situated practice communities. Individuals commence as members at the periphery of their community, develop and demonstrate relevant knowledge and skills through participating in practice, and eventually progress towards acceptance as legitimate, full members. As such, the community sanctions permitted knowledges, practices and identities, which enable these to be replicated. Full membership then, reflects the desire of individuals to participate, but also relies on individuals being legitimised and accepted by the internal hierarchy of the community (Davies, 2005). This necessitates individuals to undertake a process of becoming a certain *kind of person -*a full participant, where their fundamental identity within the community is also (re)constructed (Lave & Wenger, 1991).

These dynamics of participation, legitimacy and hierarchy, as presented in LPP, provide a framework to describe CALD practitioners’ experiences within their practice communities. This scoping review study therefore aims to review the literature regarding the experiences of CALD practitioners working within health practice contexts, using LPP as an explanatory framework.

## Methods

### Study design

Scoping review methodology was considered appropriate for this study due to the diverse knowledges and positionalities related to the research aim, and the varied nature of the literature (Thomas et al., 2020). The grey literature was considered particularly important, as a means of gathering first-person accounts of CALD health practitioners, and to reduce the impact of publication bias borne from structures that preference dominant culture perspectives (Mulimani, 2019). A scoping review was conducted, informed by the frameworks described by Levac et al. (2010) and Arksey and O’Malley (2005). This review further drew on the work of Westphaln et al. (2021) in using a team-based, mixed-methods approach, and in applying a theoretical approach, using LPP to interpret the data. The research team were from the health professions of radiography, nursing and speech-language pathology; and were from Persian, Chinese and Anglo-Australian backgrounds. In this scoping review, the sociocultural theory of Legitimate Peripheral Participation was used as a theoretical and reflexive heuristic that helped to centre the subjectivist worldview of each researcher (Chambers et al., 2018; Meyer & Ward, 2014; Thomas et al., 2020), and purpose the knowledge synthesis within a critical sociocultural framework. This use of theory enabled the research team to examine and interpret the literature for concepts related to participation, legitimacy and hierarchy; an approach observed in other scoping review studies that have critically explored concepts of cultural safety, structural racism and healthcare disparities, where applying theory, paired with a reflexive approach, enables a critical methodology that can also produce knowledge discovery (Chambers, et al., 2018; Marcewicz, et al., 2022; Zupan et al., 2021). In this review, the use of theory also enabled the research team to challenge their biases, borne from their situation within the very healthcare structures that were being examined.

Inclusion and exclusion criteria were informed by related literature, intending to capture articles describing the experiences of qualified, practicing health practitioners of CALD background as shown in Table 1. The search terms were intentionally broad, aiming to capture empirical and review studies, and grey literature (Levac et al., 2010). To represent contemporary practice experience, literature published prior to 2000 was excluded, but the language and/or country of publication was not limited. Health professions that did not require a minimum Bachelor, three year qualification were excluded, to ensure the ‘healthcare assistant’ and enrolled nurse or carer populations were not captured, as the research team perceived that power relations may impact this population differently to those in healthcare professions. To capture experiences broadly, there were no limits applied to the nature of the reported experiences, CALD health practitioner’s years from qualification, or their practice environment(s). This review did not explore the experiences of First Nations peoples, who have distinct historical and contemporary contexts that are imperative to recognise in order that they are effectively addressed (Pham et al., 2021). CALD populations were identified according to the a) ethnic groups sorted according to majority status categories in ‘The World Factbook: Field Listing—Ethnic groups’ (Central Intelligence Agency, n.d.); and where included literature indicated that participants’ language differed to the preferred language of the healthcare setting.

**Table 1:** Inclusion and Exclusion Criteria

Included	Excluded
CALD health professional/practitioner experiences	CALD students or patients
Empirical or review studies (if containing experiences of CALD practitioners which otherwise fit inclusion criteria)	First Nations health professionals and students
Grey literature (including commentaries, letters to the editor, narrative reviews from the perspective of CALD practitioners)	Published prior to 2000
	Sub-Bachelor level professional qualifications (e.g. carers, enrolled nurses)
	Articles about patient presentations, pathologies or workplace initiatives
	Systematic Reviews
	Academic Faculty
	Studies conducted in the context of war or political conflict
	Observational studies where the subjective perspective is not clearly from a CALD practitioner

### Search strategy

Search terms as shown in Table 2 and Appendix A were co-constructed with a medical librarian, and the search executed with Medline, Scopus, Emcare and CINAHL databases. The first 200 articles were screened as a pilot to test and refine the search strategy. The primary search was conducted in May 2021, and updated in August 2022. Final search results were imported into Endnote (Clarivate, 2011) and subsequently into Covidence (Veritas Health Innovation, 2021) and duplicates removed. A search for the grey literature was completed in Google, using analogous search terms and limited to the first 200 results (Haddaway, et al., 2015). Cross referencing the results identified that the grey literature yield was entirely represented in the database search, as the article type was not restricted in the search strategy, and a differentiated study selection procedure was not required for the grey literature.

**Table 2:** Search Terms

Concept 1	Concept 2	Concept 3
CALD OR “Cultural* and Linguistic* Diverse” OR Multicultural OR Multilingual OR “Ethnic* Divers*” OR BAME OR minority OR ESL OR “language other than English” OR “English as second language” OR “Black Asian minority ethnic”	“Health professional” OR “allied health professional” OR nurs* OR doctor* OR physician* OR physiotherap* OR surgeons OR midwives OR radiographers OR radiology tech* OR “speech pathologists” OR “occupational therapists” OR “healthcare providers” OR Dieticians OR audiologists OR “hearing therapists” OR “social workers” OR “allied health assistants” OR Psychologists OR Paramedics OR “emergency medical technicians” OR EMT	Experience* OR perception* OR “identity formation” OR “identity development” OR beliefs OR views OR thoughts OR feelings OR “professional identity” OR ideas OR attitudes

### Study selection

**Fig. 1:**
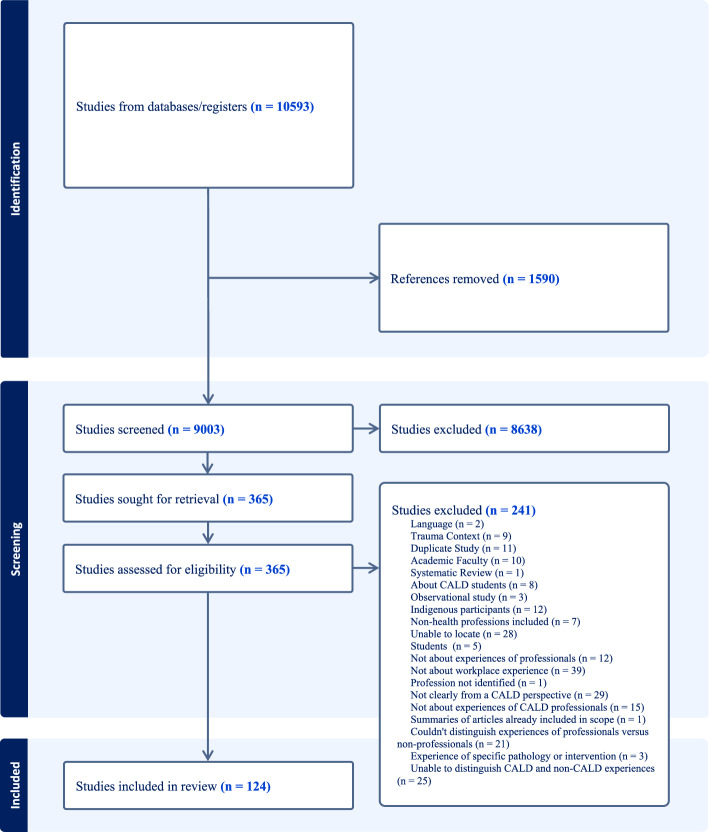
PRISMA chart

Abstract screening was conducted by two independent reviewers. Conflicts (n = 303) were discussed by the reviewers (Levac et al., 2010), and inclusion and exclusion criteria further refined. The search results represented in the Fig. 1 PRISMA chart yielded 337 articles for full text review, which then excluded a further 223 articles. Snowball sampling identified two additional articles, and one of these was included. There were 124 articles included for data extraction.

### Charting, summarising and synthesising the data

Demographic and methodological data were extracted from the included articles and analysed with descriptive statistics to broadly describe the data. Qualitative coding of the results from included studies was conducted using the conventions of Kiger and Varpio (2020) which guided textual familiarisation, coding, reviewing and defining the themes in accordance with the research question. The first author initially re-read each of the included articles, recording memos and reflective notes about how the text addressed the research question. Qualitative data that were identified as describing experiences of CALD health practitioners were then inductively coded to ensure the focus of qualitative analysis remained centred on CALD health practitioners, particularly as some articles included both CALD participants and those from the dominant culture. Each passage of relevant literature was read and annotated with a ‘code’ and grouped into categories to derive broader patterns of meaning. This was an active process whereby the first author undertook interpretation and analysis to order the data in a way that derived meaning and answered the research question, and the research team then generated and discussed potential themes in order to gain consensus and ensure that they accurately represented the coded data (Kiger & Varpio, 2020). Themes were then cross-referenced with the raw data extracts to ensure they were grounded in the data. Themes were subsequently theoretically interpreted using LPP which was operationalised into the concepts of legitimacy, peripherality and participation. These core concepts of LPP were used as a heuristic to determine if the theory explained the results (Lave & Wenger, 1991; Meyer & Ward, 2014). Finally, patterns and relationships to the theory were described to synthesise conclusions in line with the research aims.

This research was conducted in Australia, by a female research team including CALD and white-Anglo members. The research team collaborated to interpret literature that were expressed through different health professional, geographical and cultural contexts, however, this was limited by the team’s knowledge about the population/s of the regions referred. To identify CALD groups, and in interpreting the data, the team referred to The World Factbook: Field Listing—Ethnic groups’ (Central Intelligence Agency, n.d.), and took a reflexive position to examine and bracket potential cultural and professional power relations, and to sense-check the data analysis from multiple perspectives. Nonetheless, we acknowledge that bias, and the values of own practice communities may have influenced the findings. The findings were presented in a multidisciplinary research forum that included a range of student and qualified health practitioners from nursing and allied health (including several participants from CALD backgrounds), and participants from a range of professions provided feedback that the findings helped to explain their experiences. The two CALD practitioner researchers (second and third authors) further verified that the themes aligned with their understanding of their own practice experiences.

## Results

The literature yielded was prominently from English speaking countries, most frequently from the United States and Britain, followed by Australia (Table 3), using qualitative methodologies that gathered data via semi-structured interviews (Table 4). The grey literature was prominently represented by commentary and opinion pieces authored by CALD practitioners. Most articles investigated broad workplace experiences (n = 41) or multiple workplace features (n = 19), with others investigating experiences of discrimination (n = 16), career progression (n = 14), mentoring (n = 1) and professional identity formation (n = 1), and these concepts were strongly represented in the themes.

**Table 3:** Country study undertaken in and number of articles

Country	Number of Articles
United States	50
United Kingdom	35
Australia	14
Not stated	8
Canada	7
South Africa	1
Saudi Arabia	2
United Arab Emirates	2
Austria	1
New Zealand	1
Israel	1
The Netherlands	1

**Table 4:** Study design, number of articles and citations

Design	Number of studies	Citations
Qualitative	52	Mapedzahama et al. (2012), Nunez-Smith et al. (2007), Nunez-Smith et al. (2008), Likupe (2015), Esterhuizen et al. (2013), Liebschutz et al. (2006), Ho and Coady (2018), Wingfield and Chavez (2020), Etowa et al. (2009), McKenzie-Mavinga (2005), Wright and Carrese (2003), Ulloa et al. (2018), Ali and Johnson (2017), Crawford et al. (2016), Mbarushimana and Robbins (2015), O’Neill (2011), Odusanya et al. (2018), Alexis et al. (2006), Ncube (2017), Isaac (2020), Lee et al. (2010), Estacio and Saidy-Khan (2014), Matza et al. (2018), Alexis and Shillingford (2015), Ko and Dorri (2019), Philip et al. (2019), Cottingham et al. (2018), Engstrom et al. (2009), Almutairi et al. (2015), Das (2020), Harrison (2007), Hagey et al. (2001), Lévesque and Negura (2021), Xiao et al (2014), Terry et al. (2013), Simpson and Ramsay (2014), Frimpong (2016), Qureshi et al. (2020), Le and Kilpatrick (2008), Tanega-Doster (2011), Collins (2004), Cohen (2013), Song and McDonald (2021), Henry (2007), Iordan (2018), Cottingham and Andringa (2020), Lim et al. (2022), Tedam (2021), Chang et al. (2021), Beagan et al. (2022), Chen et al. (2010)
Grey literature	29	Olayiwola (2016), Pickersgill (2004), Charleston et al. (2020), Sinkfield-Morey (2019), Anderson (2020), Champion (2021), Phillips (2004), Myers (2011), Duffin (2012), Coghill (2013), Jones (2013), Jones-Berry (2016), Anionwu (2017), Oshikanlu (2020), Newland (2020), Handley (2015), Trueland (2012), deFigueiredo (2011), Cooper et al. (2008), Gould & Kirkley (2017), Hu (2016), Agnew (2019), Argueza et al. (2021), El Gindy (2006), Villarruel (2017), Lewenson and Graham-Perel (2020), Cowan et al. (2021), Foong (2022), Waldman (2000)
Quantitative	17	Oladeji et al. (2018), Timilsina Bhandari et al. (2015), Nunez-Smith et al. (2009), Alshehry et al. (2019), Brown et al. (2009), Erhunmwunsee et al. (2019), McGinnis and Moore (2009), Ying (2015), Criddle et al., (2017), Gasiorek and Van der Poel (2018), Arriaza (2015), Morris et al. (2011), Teran et al. (2017), Robinson et al. (2020), Johnson et al. (2021), Dyrbye et al. (2022), Samora et al. (2020)
Literature review	6	Robinson (2013), Cortés-Guiral et al. (2021), Likupe (2006), Tuttas (2015), Snyder and Schwartz (2019), Pendleton (2017)
Cross sectional	6	Abu Rabia et al (2021), Agrawal et al. (2018), Wyatt et al. (2021), Sudol et al. (2021), Connor and Miller (2014), Ruzycki et al. (2023)
Not stated or unclear	3	Kelly and Greene (2010), Pedrotti and Burnes (2016), Nezu (2010)
Phenomenology	3	Brooks (2016), Alexis and Vydelingum (2005), Wros (2009)
Mixed methods	2	Tan and Denson (2019), Holmes and Grech (2015)
Ethnography	2	Iheduru-Anderson (2020), Yeowell (2013)
Grounded theory	2	Deegan and Simkin (2010), Yan (2008)
Autoethnography	1	Wang et al. (2020)
Scoping review	1	Zhong et al. (2017)

The experiences of CALD nurses (n = 66) and medical practitioners (n = 30) were most frequently reported (78% of included articles). Other health professions included psychology (n = 11), social workers (n = 6), dentistry (n = 1) and physiotherapy (n = 1). Four articles reported the experiences of practitioners from multiple professions.

A range of racial/ethnic groups were represented across professions (Table 5), and some articles included participants from different backgrounds, including articles that included both participants from CALD backgrounds and those from the dominant culture. Table 6 shows that Asian practitioners from a range of countries were most frequently included (n = 42), followed by North American (n = 31) and African (n = 30) participants. However, much of the included literature did not attribute any country of origin (n = 64) or ethnicity (n = 18). Articles prominently explored the adjustment experiences of immigrant CALD practitioners (n = 119) and less frequently reflected experiences of practitioners who were from a racial, ethnic or cultural minority population in their home country (n = 37). Over a third of studies about practitioners who were from a racial or cultural minority group in their home country included participants from multiple racial groups (n = 14).

**Table 5:** Participant racial/ethnic background across professions

Profession (n = 124)	Race/Ethnicity n (%)	
Social work (n = 7)	Multiple	3 (42.9)
	Not stated/unsure	3 (42.9)
	Black	1 (14.3)
Medicine (n = 30)	Multiple	13 (43.3)
	African	3 (10)
	African America	3 (10)
	Note stated/unsure	3 (10)
	Other	3 (10)
	Asian	3 (10)
	Black	1 (3.3)
	Indian	1 (3.3)
Occupational therapy (n = 1)	Multiple	1 (100.0)
Dentistry (n = 1)	Multiple	5 (45.5)
	Black	1 (9.1)
	African American	1 (9.1)
	Not stated/unsure	2 (18.2)
	Other	2 (18.2)
Psychology (n = 11)	Multiple	1 (100.0)
Physiotherapy (n = 4)	Black	1 (25.0)
	Multiple	1 (25.0)
	Other	2 (50.0)
Nursing (n = 64)	African	4 (6.0)
	African American	3 (4.5)
	Asian	1 (1.5)
	Black	14 (20.9)
	Multiple	21 (31.3)
	Not stated/unsure	8 (11.9)
	Other	16 (23.9)
Multiple professions (n = 1)	Not stated/unsure	1 (100.0)
Other (n = 2)	Black	1 (50.0)
	Multiple	1 (50.0)

**Table 6:** Reported ethnic and cultural groups

Continent of origin of ethnic group	Total number of articles	Race and cultural groups represented in articles
Africa	30	African, South African, Sub-Saharan African, Ghanian, Zimbabwean, Black African, Kenyan, Zambian, Jamaican, African Congolese,
Asia	42	Filipino, Indian, Chinese, South Asian, Vietnamese, Taiwanese-Chinese, Nepalese, East Asian, Singaporean, Laotian, Asian Indian
Northern America	31	African American, Caribbean, Japanese American, Black Caribbean, Anglo American, Chinese American, East Indian American, African Caribbean, Afro Caribbean, Caribbean American
Southern America	23	Hispanic, Latino, Latin American, Latinx, Other Spanish Origin, El Salvador
Europe	14	European, British South Asian, Indian-British, British-Asian, Black British, Black British Ghanian, Black British African, British, Czechoslovakian, French, German-Polish, Danish-Dane, Eastern European
Middle East	6	Middle Eastern, Iranian, Bedouin
Australia and Oceania	2	Australian, New Zealander
Not regionally defined	64	Black, Asian, BME, White, Non-Hispanic White, Muslim, Black-White-Biracial, White Bicultural, Jewish, Non-Hispanic Black, Non-Hispanic Asian, Non-Hispanic Other, People of Colour, Biracial
Ethnic group unstated	18	

### Qualitative themes

### Theme 1: discrimination

As the most prominent theme, ‘Discrimination’ captured the overarching experiences of CALD practitioners, whilst also relating to the two remaining themes of ‘Consequences’ and ‘Hierarchies’. Three subthemes comprised the ‘Discrimination’ theme: ‘Overt Discrimination’, ‘Covert Discrimination’ and ‘Barriers to addressing Discrimination’.

#### Overt discrimination

CALD practitioners described discrimination as occurring overtly and stemming from several workplace sources. Discriminatory experiences with patients often took the form of refusal of care due to the practitioner’s race or other identifiers (Frimpong, 2016; Nunez-Smith et al., 2007; Tuttas, 2015) extending to racist remarks and harassment (Cabral & Smith, 2011; Mbarushimana & Robbins, 2015; Wingfield & Chavez, 2020). Experiences of overt discrimination from patients were reported in settings where the practitioner was treating white patients (Wingfield & Chavez, 2020), those with mental health (Cottingham et al., 2018) or drug and alcohol problems, such as in emergency departments (Frimpong, 2016; Wingfield & Chavez, 2020).

‘He was a very difficult patient in that he was racially and sexually inappropriate with staff... he called me a “nigger,” a “humpbacked monkey” several times.’ (Cottingham et al., 2018, p150)

Several studies reported experiences of overt discrimination from colleagues and supervisors including verbal assault, marginalisation and bullying due to race, culture or communication (Frimpong, 2016; Lewenson & Graham-Perel, 2020; Tuttas, 2015; Zhong et al., 2017), and instances of colleagues leaving or refusing to work alongside a CALD practitioner (Lewenson & Graham-Perel, 2020; Newland, 2020). In one study, more than 40% of CALD nurses perceived some form of racial harassment from colleagues (Tuttas, 2015).

‘The most painful discrimination occurred in the forms of jokes to demean Africans. “I like to laugh with people, but not at the expense of others; how do you laugh when a colleague shouts ‘Ebola!’ as you walk towards him in a hospital?”’ (Frimpong, 2016 p76)

#### Covert discrimination

Covert discrimination was reported as including perceived resentment and distrust from colleagues (Mapedzahama et al., 2012) feeling unsupported in decisions (Frimpong, 2016) being ‘surveilled’, excessively watched and monitored by peers and supervisors (Mapedzahama et al., 2012; Xiao et al., 2014) with an undercurrent of fault finding (Cottingham et al., 2018; Holmes & Grech, 2015), and unfair or undue criticism or complaint from colleagues (Alexis & Vydelingum, 2005; Pedrotti & Burnes, 2016). This scrutiny resulted in stress that impacted performance of CALD practitioners (Holmes & Grech, 2015; Mapedzahama et al., 2012), described as ‘perpetuating the myth of incompetence’ (Mapedzahama et al., 2012 p157).

‘[workmates] will be following you like they don’t trust what you are doing, or they can even ask you … a question which really annoys like: Can you do blood pressure? Obviously! How could you ask that question? And that person will repeat, keep asking you the same question!’ (Mapedzahama et al., 2012 p160).

In some studies, CALD practitioners reported being subjected to unfair treatment or unequal workload distribution, for example being allocated difficult patients, working overtime or unpopular shifts (Alexis & Vydelingum, 2005; Frimpong, 2016), and lacking acknowledgement despite undertaking the same or additional workload as their peers (Alexis & Vydelingum, 2005; Tuttas, 2015). CALD practitioners were further marginalised through unspoken rules and group norms that contributed to their lacking confidence with social and ‘lunchroom’ interactions, This resulted in…*’an overwhelming sense of isolation and separateness from… colleagues’* (Frimpong, 2016, p72) that was exacerbated if they lacked experience with informal English (Xiao et al., 2014).

CALD practitioners reported experiencing microaggressions founded in unacknowledged racialised stereotypes relating to their inferiority or ‘backwardness’, or assumptions about their educational background and (in)competence (Cottingham et al., 2018; Nunez-Smith et al., 2007). Black practitioners reported being perceived as ‘lazy’, ‘not serious’ (Wingfield & Chavez, 2020) p43, ‘stupid’ (Frimpong, 2016) p78 or from underdeveloped nations (Mapedzahama et al., 2012).

‘Some of them [have] no clue … they think we came from trees, that we used to live with monkeys, and then we just got off the tree and boarded an aeroplane and came here.’ (Mapedzahama et al., 2012) p158

Other microaggressions reflected covert behaviours that generated discomfort for the CALD practitioner, including instances of colleagues using racial slurs in their presence (Wingfield & Chavez, 2020), or asking culturally inappropriate questions (Tuttas, 2015).

#### Barriers to challenging discrimination

Denial, acceptance and the minimisation of workplace discrimination presented barriers for CALD practitioners to challenge discrimination (Mbarushimana & Robbins, 2015; Nunez-Smith et al., 2007; Pedrotti & Burnes, 2016; Tuttas, 2015). The literature identified workplace actions that conveyed an underlying acceptance and condonation of discrimination.

'I was a triage nurse in the emergency room for many years and the nurse manager would transfer patients to other nurses when patients openly said they wouldn’t allow the African nurse to triage them' (Frimpong, 2016, p78).

The absence of clear organisational policy or procedures presented additional barriers to challenging discrimination (Mbarushimana & Robbins, 2015; Nunez-Smith et al., 2007), further compounded as CALD practitioners reported uncertainty about how to manage covert or less explicit discrimination that was difficult to prove (Frimpong, 2016). Conversely, some articles reported CALD practitioners resisted actions they perceived would result in negative repercussions. For example, one medical practitioner reported that challenging discrimination would *‘get him in trouble’* (Anderson, 2020, p1610) and described experiences throughout his career of being warned of marginalisation for speaking out (Anderson, 2020). Similarly, multiple studies about immigrant health practitioners described visa and financial pressures as barriers to challenging discrimination (Pedrotti & Burnes, 2016; Tuttas, 2015; Xiao et al., 2014), and these factors also enabled exploitative working conditions (Tuttas, 2015).

‘The first priority for immigrant nurses is to survive and gain employment. As a consequence they are often more accepting of ‘discrimination’, less likely to be assertive, or critical of their peers, or to take on leadership roles.’ (Xiao et al., 2014, p646)

The impacts of discrimination and the challenges for CALD practitioners in acting on or removing themselves from such experiences were situated within a struggle to be recognised as having legitimacy in contributing to their healthcare settings. These experiences often manifested in the marginalisation of CALD practitioners, who were not always fully accepted as healthcare members.

### Theme 2: consequences

The theme ‘Consequences’ captured the cognitive, emotional, behavioural, interpersonal and self-perceptual adjustments that CALD practitioners enacted through experiencing discrimination; or transitioning into workplaces. These adjustments are broadly categorised into ‘professional identity’, and ‘behaviours and coping strategies’.

#### Professional identity

In this study, professional identity is used to describe internal changes that were reported to occur as a consequence of CALD practitioners’ experiences. Many of these changes impacted negatively, however, positive adjustments that empowered individuals within their workplace were also identified.

CALD practitioners often reported that workplace discrimination impacted their self-perception as practitioners. Multiple studies reported affective consequences, including decreased self-efficacy (Cottingham et al., 2018; Pedrotti & Burnes, 2016; Tuttas, 2015), increased self-doubt and rumination (Beagan, et al. 2022; Cottingham et al., 2018), erosion of confidence (Zhong et al., 2017) and feelings of inadequacy (Alexis & Vydelingum, 2005). Studies also reported that CALD practitioners who continued to work in the context of discrimination experienced internal conflict and emotional exhaustion that interfered with their work (Beagan, et al., 2022; Cottingham et al., 2018; Pedrotti & Burnes, 2016).

‘“I’ve encountered four instances of systemic racism and it’s only eleven o’clock in the morning. How do you think I’m doing?”…Really, really heavy… I am currently in a space of moral fatigue’ (Beagan, et al. 2022, p7).

These feelings of inadequacy and low self-efficacy were partnered with perceptions of needing to ‘prove’ oneself to colleagues and make additional efforts to be accepted (Mbarushimana & Robbins, 2015; McKenzie-Mavinga, 2005; Pedrotti & Burnes, 2016).

‘I have to think that I will one day I will be right there. I have to prove myself that I can be one of them’ (Alexis & Vydelingum, 2005, p469).

Experiences of discrimination negatively impacted CALD practitioners’ perceptions of their profession, their work and job satisfaction (Pedrotti & Burnes, 2016; Ying, 2015; Zhong et al., 2017), and were associated with thoughts of leaving the profession (Frimpong, 2016) or returning to home country (Zhong et al., 2017).

‘Chinese nurses… got stuck in their career, failed to fit into the host community, submitted to their families’ demands, or simply filled with longing for home, where they were not identified as outsiders’ (Zhong et al., 2017, p108).

Conversely, in some studies CALD practitioners reported satisfaction from aspects of their work that were less enjoyed by their peers. In particular, they derived meaning from the intercultural environments in which they worked (Mbarushimana & Robbins, 2015), in utilising culture and language skills (Hu, 2016), or engaging with patients from minority backgrounds (Nunez-Smith et al., 2007). In these aspects, CALD practitioners identified themselves as assets to the healthcare team (Xiao et al., 2014).

‘Being a member of that culture gave me insights to handle that situation in a culturally sensitive manner.… Serving the Chinese-speaking community has given me much fulfillment. It is an obligation that I feel in my heart.’ (Hu, 2016, p200).

#### Behaviours and coping strategies

CALD practitioners developed behaviours and coping strategies to respond to discrimination. These adjustments were empowering or disempowering, either facilitating the contesting of barriers and stereotypes, or the acceptance of discrimination and struggle.

CALD practitioners reported using strategies that increased their control or agency, that were ‘empowering’ despite compromising features of their cultural, racial or gender identity (Anderson, 2020; O’Neill, 2011) as they facilitated their ‘fit’ within their professional communities. These strategies included using formal professional attire and titles (Anderson, 2020; Pedrotti & Burnes, 2016), altering communication and speech (Almutairi et al., 2013; Zhong et al., 2017), adapting to cultural expectations of professionalism, such as being chatty with patients (O’Neill, 2011), or modifying public behaviours to align with western values (Zhong et al., 2017).

CALD practitioners also generated professional support networks, including subcultures of similar racial, cultural and language groups within their workplaces to manage their exclusion (Beagan, et al., 2022; Xiao et al., 2014) and protect their cultural identity (Alexis & Vydelingum, 2005). These informal networks were noted across nursing and medical, and allied health professions enabling CALD practitioners to cope with discrimination when institutional support was lacking (Alexis & Vydelingum, 2005; Beagan, et al., 2022; Cottingham et al., 2018; Duffin, 2012; Lewenson & Graham-Perel, 2020; Nunez-Smith et al., 2007; Pedrotti & Burnes, 2016).

BME (black and minority ethnic) colleagues also supported each other in carrying out their work… to say, you know, ‘This is what I’m being faced with. What would you advise?’’ (Mbarushimana & Robbins, 2015, p147).

Several studies reported career changes as empowering CALD practitioners to manage discrimination or leave unsupportive workplaces. Whilst career changes were often perceived negatively, they empowered individuals to reclaim agency (Anderson, 2020; Nunez-Smith et al., 2007).

‘I transferred from the emergency room to intensive care after my first year because I couldn’t tolerate the discrimination in the department. It came from the patients, EMT workers and physicians.’ (Frimpong, 2016, p78).

CALD practitioners were also empowered through intentionally ignoring or disregarding discrimination to minimise its burden and reclaim control (Cottingham et al., 2018; Nunez-Smith et al., 2007), actively challenging discrimination by speaking out or educating those around them (Anderson, 2020; Beagan et al., 2022; Mbarushimana & Robbins, 2015), or making conscious efforts to highlight their strengths (Hu, 2016; Mapedzahama et al., 2012).

‘I make a point…of interjecting the importance of race and culture and cultural competence in how we interface with patients especially’ (Nunez-Smith et al., 2007 p48).

However, CALD practitioners reported being disempowered when they were unable to express or manage the consequences of discrimination (Almutairi et al., 2015; Frimpong, 2016; Holmes & Grech, 2015; O’Neill, 2011), with some employing strategies to cope that resulted in professional isolation (Frimpong, 2016) or withdrawing to subordinate roles (Holmes & Grech, 2015; O’Neill, 2011) that impacted their self-identity.

‘Some relinquished responsibilities and critical thinking skills, a safe position where the risk of communication breakdown would be minimised.’ (O’Neill, 2011, p1124).

CALD practitioners also reported physical and psychological consequences of workplace discrimination, manifesting in sleep problems, weight loss and chest pain (Holmes & Grech, 2015) and psychological disturbances (Zhong et al., 2017).

The categories that described the Consequences reflected the peripherality of the position of many CALD practitioners in their practice communities. Consequences and strategies could further distance CALD practitioners from others in their workplace, or adaptively respond to better ‘fit’ into their community.

### Theme 3: hierarchy

The final theme identified racial hierarchies that existed within and between health professions, where some professions and individuals were ascribed to hold more power and status than others. These concepts intersected, as CALD practitioners reported their experiences as influenced by both professional status and race.

#### Racial hierarchy

Intersections between professional hierarchies and CALD identity were often reported (Almutairi et al., 2015; Cottingham et al., 2018; McGinnis & Moore, 2009; Nunez-Smith et al., 2007; Tuttas, 2015). For example, CALD nurses reported more frequent experiences of overt discrimination than CALD doctors, regardless of their gender (Agrawal et al., 2018; Nunez-Smith et al., 2007; Wingfield & Chavez, 2020). The concept of hierarchy also captured inter-racial power differences, that elevated career and work opportunities for ethnic groups with high status, and resulted in further experiences of discrimination for groups with lower status (Almutairi et al., 2015; Frimpong, 2016; Tuttas, 2015).

'Some nationalities also look down on the Filipinos as if they are stupid, less knowledge like that'. (Almutairi et al., 2015, p 21).

CALD practitioners across professions reported stereotypical associations about the nature of their role, skills and work, including assumptions that they were employed as cleaners, unregistered nurses or healthcare assistants (Cottingham et al., 2018; O’Neill, 2011; Pedrotti & Burnes, 2016; Wingfield & Chavez, 2020).

‘As a black physician you keep getting mistaken for other people….Some of my white female [colleagues] were always getting mistaken for nurses and everyone thought that was funny. But I was always mistaken for the person who brought the trays or the janitor person. So I think the things for which they mistake us is much more disparate for black women.’ (Nunez-Smith et al., 2007, p48).

Several articles reported that CALD practitioners’ qualifications, experience and their professional skills were under-valued compared with their peers (de Figueiredo, 2011; Mbarushimana & Robbins, 2015; Tuttas, 2015; Xiao et al., 2014; Zhong et al., 2017). Career progression was impeded by bureaucracy that prevented skill utilisation (Alexis & Vydelingum, 2005; de Figueiredo, 2011) and marginalisation or lack of promotion (Alexis & Vydelingum, 2005; Johnson, et al., 2021; Tuttas, 2015). Racial hierarchies were reported in hiring and recruitment structures (Tuttas, 2015; Wingfield & Chavez, 2020; Yeowell, 2013).

‘There is no person in leadership or management or a VP role of a visible minority. None. Not one manager. So I’m pretty angry at that. I’m angry at the fact that they won’t even acknowledge that that’s a problem.’ (Beagan et al., 2022, p6).

These hierarchies limited leadership opportunities afforded to CALD practitioners (Anderson, 2020; Duffin, 2012; Wingfield & Chavez, 2020), underpinning pay differences, where white followed by Asian health practitioners were reported as earning more than Hispanic and Black health practitioners despite equivalent education and experience (McGinnis & Moore, 2009; Waldman, 2000), and white university graduates were preferred over CALD graduates (Wingfield & Chavez, 2020). However, CALD practitioners also reported feeling obliged to employers who provided them work opportunities, rendering many unable to discuss issues of workplace discrimination (Mbarushimana & Robbins, 2015).

Across the health professions, CALD practitioners reported being funnelled into ‘diversity’ related workloads or unfulfilling career pathways (Anderson, 2020; Erhunmwunsee et al., 2019; Mbarushimana & Robbins, 2015; Pedrotti & Burnes, 2016). This was likened to a ‘*cultural taxation*’ whereby the practitioner experiences increased workload or burden as an ‘*ambassador*’ for their cultural group (Pedrotti & Burnes, 2016 p145), leading to reflections of being ‘*just a diversity statistic?*’(Anderson, 2020 p1610). Whilst these interventions often intended to address workplace disparities, or shine light on the skills that CALD practitioners bring to diverse populations, they may also perpetuate existing hierarchies.

‘Participants also described being involuntarily “cast” into race-based roles in the workplace… include[ing] helping with minority physician recruitment, serving on numerous diversity committees, intervening in difficult situations with minority colleagues or trainees, and assisting nonminority colleagues in the care of minority patients.’ (Nunez-Smith et al., 2007 p48).

#### Professional and gender hierarchies

Intersections between professional hierarchies with gender-based power imbalances were prominently reported in the medical and nursing professions, and this amplified experiences of discrimination. The character traits associated with medicine were considered masculine, i.e. including competence, intelligence and strength, that were protective against discrimination.

“It is hard being a physician of color because you have the issue of race and the issue of power. When you are a physician, you have a power position that other people don’t have, whether they are of the same race or different race or whatever” (Nunez-Smith et al., 2007) p47.

Conversely, in professions considered feminine, including nursing, societal stereotypes characterise deference, submission and caring, that can generate perceptions of a lack of professionalism (Shannon et al., 2019; Wingfield & Chavez, 2020). In nursing, male CALD nurses have reported a ‘glass escalator’ effect in which they identified greater likelihood of promotion to leadership positions (Frimpong, 2016). Conversely, female CALD nurses reported greater frequency of discrimination, experiencing the ‘emotional double shift’ of race and gender (Cottingham et al., 2018). As such, having a high-status profession and being male gender may have a protective effect from discrimination experiences for CALD practitioners.

The theme of hierarchy embodies notions of ‘power over’ that for CALD practitioners relate inherently to their capacity to participate in the activities of their practice community, be recognised as having a legitimate and valued contribution, and be enabled to progress and demonstrate leadership from the centre of the community.

## Discussion

This scoping review describes the experiences of CALD practitioners in dominant culture healthcare settings, revealing themes of discrimination, consequences, and hierarchy. The included literature was overwhelmingly derived from Western countries, in the fields of medicine and nursing, and predominantly describing the experiences of CALD practitioners from African, Asian and North American backgrounds. There was comparatively little literature pertaining to other health professions. In this discussion, the theory of Legitimate Peripheral Participation (Lave & Wenger, 1991) is used to conceptualise how CALD practitioners are facilitated or hindered in becoming legitimate members of their professional community, and the identities they inhabit to pursue this.

Discrimination was a barrier to participation and legitimacy, with CALD practitioners reporting both positive and negative personal and structural consequences. Lave and Wenger (1991) theorised that learning and integration into a practice community occurs when individuals can access legitimate opportunities to participate. Instances of overt discrimination resulted in reduced practice opportunities that maintained the position of CALD practitioners at the periphery of the community. For example, reallocating racially abusive patients from CALD practitioners reinforces and legitimises these patient behaviours and concurrently impedes opportunities for CALD practitioners to demonstrate complex practice skills, which may contribute to challenges in career progression. Experiences of covert discrimination perpetuated notions of illegitimacy and incompetence through stereotyping, negative associations, and bias (Lennartz et al., 2019; Wingfield & Chavez, 2020). Consequently, CALD practitioners reported experiencing social exclusion within their practice communities – both self-initiated and community-instigated. This positioning at the periphery of the practice community, may further legitimise negative bias in hiring, employment and career progression opportunities (Tuttas, 2015; Wingfield & Chavez, 2020; Yeowell, 2013). Conversely, affirmative action employment and progression interventions that intend to facilitate professional diversity may leave CALD practitioners feeling fraudulent or indebted for their place in their practice community (Standing & Baume, 2001). These perceptions of ‘being permitted’ entry, may ascribe CALD practitioners to tolerate discrimination and structures that hold their position at the periphery of their community.

Identity adjustments that CALD practitioners enacted in response to discrimination either enhanced their legitimacy as members of their practice community or compounded their position at the periphery. Identity formation is critical for professional socialisation, as new community members acquire the knowledge, skills and identity attributes that are both endorsed by, and permit acceptance into the community (Davies, 2005; Lave & Wenger, 1991). The structural consequences of discrimination and resulting negative self-perceptions reduce CALD practitioners’ opportunities to participate and effectively demonstrate the practice attributes required for endorsement (Davies, 2005). Conversely, CALD practitioners who adjust to fit within their professional community, such as through their masking of culturally-derived behaviours, dress and discourses may be perceived as more legitimate, as they demonstrate identity attributes aligned with the dominant culture community. Such adjustments may be rewarded with further participation opportunities that improve the position and stature of the CALD practitioner, whilst further perpetuating dominant culture practices and biases (Attrill et al., 2022). In both scenarios, this compromise of the CALD person’s values and identity to respond to the values or perceptions of the dominant community are reported as psychologically depleting, and may contribute to professional attrition (Frimpong, 2016; Nunez-Smith et al., 2007; Pedrotti & Burnes, 2016; Tuttas, 2015; Zhong et al., 2017).

The findings also suggested that some CALD practitioners derive satisfaction from serving diverse communities and often bridge cultural and linguistic barriers, verifying research that identifies CALD practitioners as potential cultural brokers with patients (Berger, 2008; Cabral & Smith, 2011; Nápoles-Springer et al., 2005). They reported their shared language and lived experiences as facilitating connection with CALD patients that elevated the quality of care (Hu, 2016; Mbarushimana & Robbins, 2015). Prior research suggests that “giving back” to CALD communities provides meaning that is foundational in constructing the professional identities of CALD practitioners (Wyatt et al., 2021). These identities appear to differ from those of the dominant practice community, and evidence exists that the skills of CALD practitioners in working with CALD patients are both valued (Xiao et al., 2014) or create further marginalisation (Mbarushimana & Robbins, 2015; Nunez-Smith et al., 2007). Lave and Wegner (1991) theorised that communities can adopt knowledges and practices of new members, and these new practices may become legitimised if they have value and are repeated through members’ participation. Celebrating and elevating the knowledge and skills that CALD practitioners contribute may inform and model culturally responsive practices for the broader practice community. This may in-turn legitimise CALD practitioners, whilst also protecting against the deleterious impacts of discrimination (Hu, 2016; Mapedzahama et al., 2012; Nunez-Smith et al., 2007). Recognition that these knowledge, skills and attributes are critical to address healthcare inequities may reduce the ‘cultural-taxation’ (Pedrotti & Burnes, 2016 p145) that CALD practitioners report in enacting their advocacy for diverse communities. This requires these behaviours to be legitimised, valued and endorsed at all levels of the community, including those who benefit from professional hierarchies and high status.

The professional and racial hierarchies identified in this study reiterate boundaries that CALD practitioners experience in participating in healthcare practice. These boundaries act to reinforce dominant culture knowledge and practices that hold CALD practitioners at the periphery of their community. Wingfield and Chavez (2020) noted that these barriers become more prominent as CALD practitioners seek increasingly senior opportunities. Davies (2005) observed that an individual’s identity within a community is mediated by their relationship with the recursive meanings and social structures of that community, and by extension, the world in which it exists. Power and internal hierarchies that present and are maintained in organisational and leadership structures may present greater identity and value conflicts for CALD practitioners, generating further barriers to their participation and success in these positions. Thus, the poor visibility of CALD practitioners in leadership is not only a *symptom* of hierarchy, but *perpetuates* hierarchy, where dominant culture practitioners who are members of high-status professions retain power, and power subsequently diminishes through intersections between professional, racial and gender hierarchies. These persistent and intersecting power structures are also visible in the education programs that CALD health professional students encounter during training, and are particularly pervasive during clinical practice training experiences (Attrill, et al., 2022; Joseph et al., 2021). In this study, as for previous research about CALD students, CALD health practitioners often demonstrated adaptive strategies within the environments in which their power imbalance was impactful, but these strategies often sought to reduce their personal impact from being discriminated against – there were few examples where adaptive responses enabled the participants’ greater personal power or success. It is likely that future strategies to address workforce diversity will need to accommodate the full trajectory from student training to workforce participation, and address power as it intersects across and through hierarchies.

Further marginalisation is likely experienced by practitioners with added disadvantage, such as through experiencing disability. These multiple intersections create new dynamics of power and forms of discrimination that cannot be addressed by attending to each element in isolation (Collins, 1990). This review suggests that these intersections compounded the CALD practitioners’ experiences of discrimination and their resultant impacts on participation and legitimation. Additionally, whilst this review was largely grounded in literature from medicine and nursing, the broader summarised literature suggests that these intersectional issues are common to other health professions, including social work (Mbarushimana & Robbins, 2015), physiotherapy (Yeowell, 2013) and psychology (Pedrotti & Burnes, 2016). Meaningful change will require these intersectional barriers that reinforce and sustain dominant health professional hierarchies and cultures to be reflexively examined with actions designed with CALD practitioners and grounded in their perspectives (Wyatt et al., 2021).

## Limitations

Clustering CALD practitioners together for this research helped to amplify their experiences in dominant culture healthcare settings. However, the cultural, ethnic, linguistic and historical backgrounds of individuals and populations included in this research are diverse and their reductive grouping inadvertently diminishes important distinguishing features, and masks differences in their needs and experiences. The intention of this review was not to diminish the experiences of particular cultural or ethnic groups, or indeed, individuals, but rather to provide evidence of their interactions within dominant practice constructs to inform practice and policy change. Whilst this review intended to amplify the perspectives of CALD practitioners, the research team acknowledge that their own subjectivity and bias may have had the affect of amplifying or quelling this through the interpretative process. The grey literature produced a large volume of first-person CALD practitioner accounts which were highly valued inclusions in this research, and we acknowledge that more extensive grey literature searching may have produced further variety of perspectives.

## Conclusion

CALD practitioners are often participating in healthcare communities whose professional identities are structured around a Western, English-speaking framework. Given the need to embody these practice-situated identities to progress and gain experiences for legitimacy, the barriers to legitimation are greater for practitioners who are not from the dominant culture. The findings of this review highlighted the structural nature of discrimination that CALD practitioners experience, and their entrenched and disempowered positions within recursive professional health hierarchies (Attrill et al., 2022). To respond, institutional policy and procedures should address the multiple, intersectional configurations of discrimination that inform the experiences of CALD practitioners. However, meaningful change can be achieved only through critically examining the structures and practices, and the dominant discourses and narratives that sustain institutional and health professional cultures and marginalise those at the periphery (Wyatt et al., 2021).

## Electronic supplementary material

Below is the link to the electronic supplementary material.

Supplementary file1 (DOCX 2146 KB)

## Figures and Tables

**Fig. 2:**
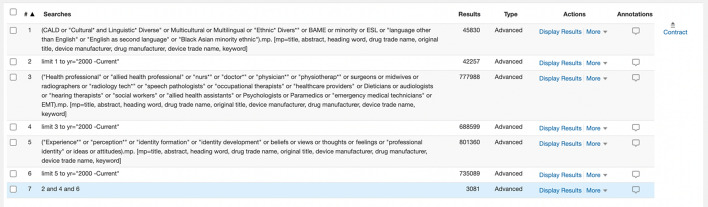
Emcare

**Fig. 3:**
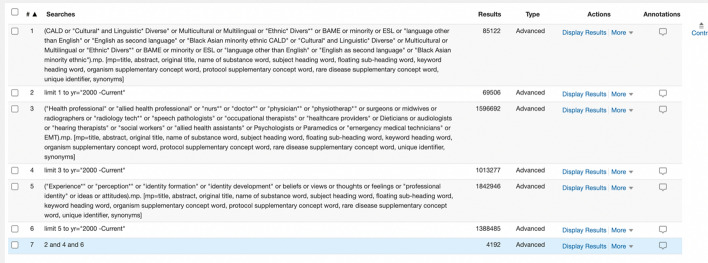
Medline

**Fig. 4:**
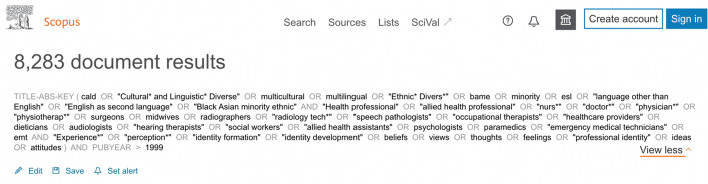
Scopus

**Fig. 5:**
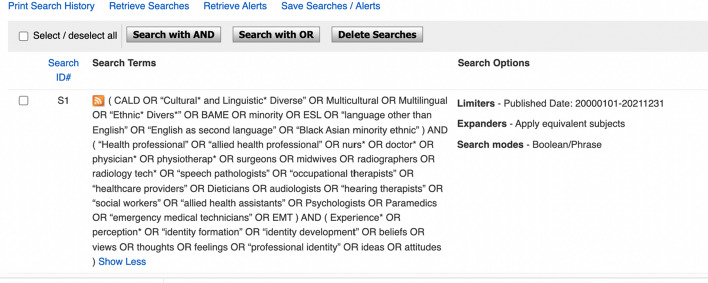
CINAHL

## Data Availability

The datasets used and/or analysed during the current study are available from the corresponding author on reasonable request.

## Appendix A: Search strategies for Emcare, Medline, Scopus and CINAHL

See Appendix Figs. 2, 3, 4 and 5
