# Supplementary material for: The experiences of culturally and linguistically diverse health practitioners in dominant culture practice: a scoping review
Source: Adv Health Sci Educ Theory Pract. 2024 Jul 22;30(2):613–43. doi: 10.1007/s10459-024-10359-7 (PMC11965172; doi:10.1007/s10459-024-10359-7)
Supplement: Supplementary file 1 — Supplementary file1 (DOCX 2146 KB) [file 10459_2024_10359_MOESM1_ESM.docx]

**Appendix A:** *Search strategies for Emcare, Medline, Scopus and CINAHL*

Figure A1*: Emcare*

*
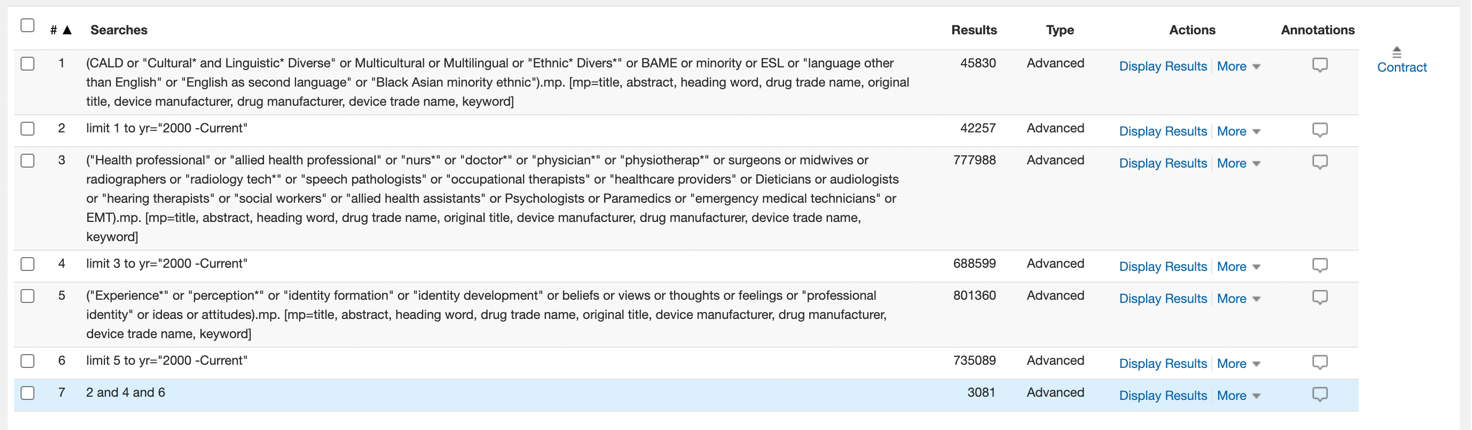
*

Figure A2: *Medline*

*
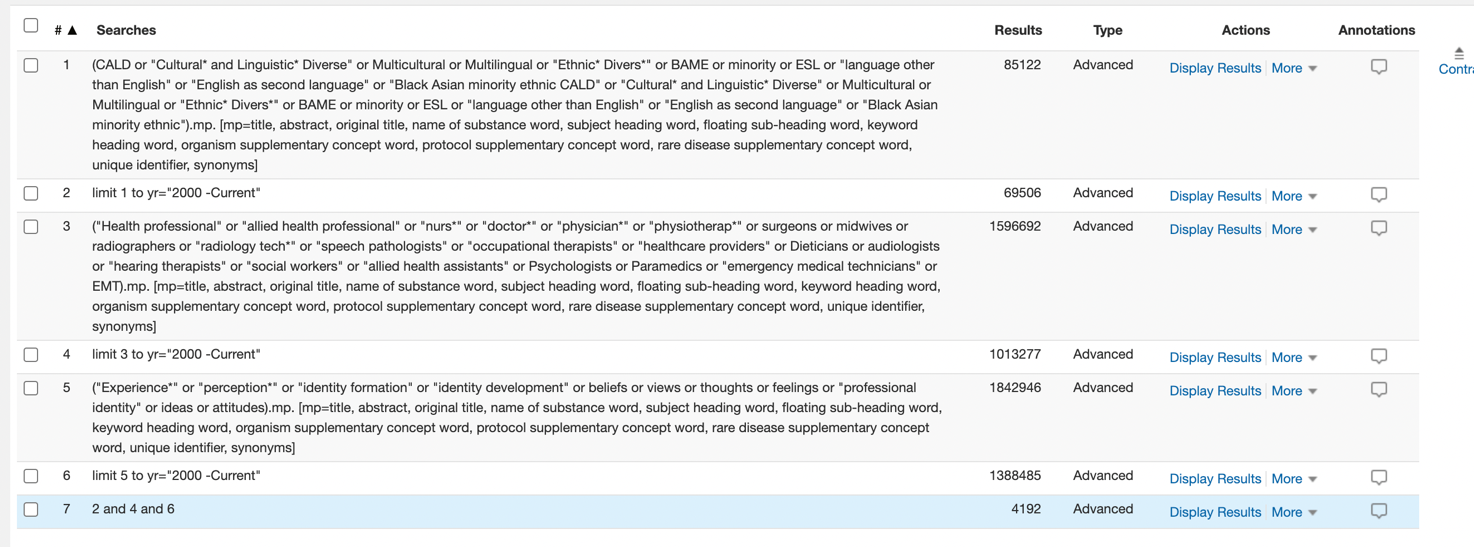
*

Figure A3: *Scopus*

*
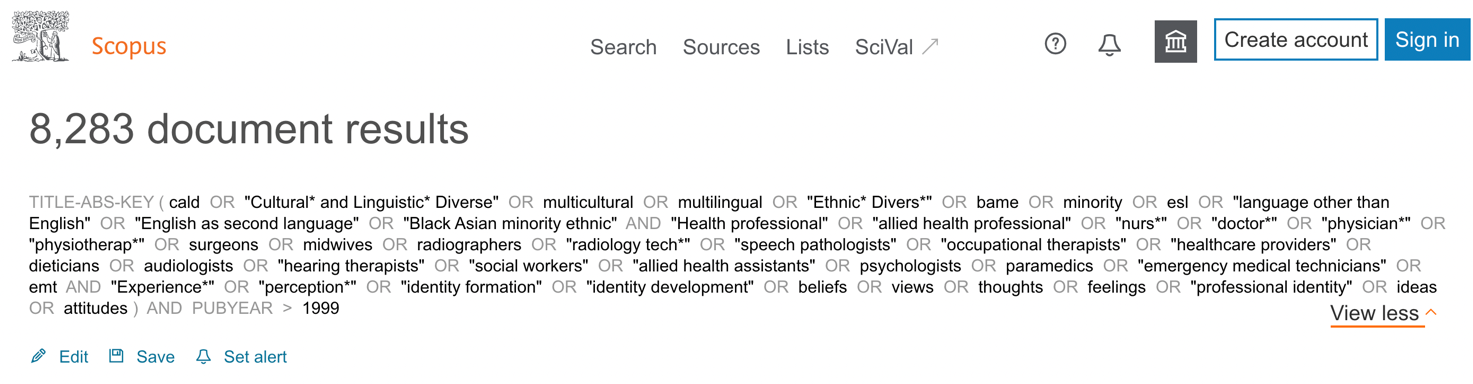
*

Figure A4: *CINAHL*

*
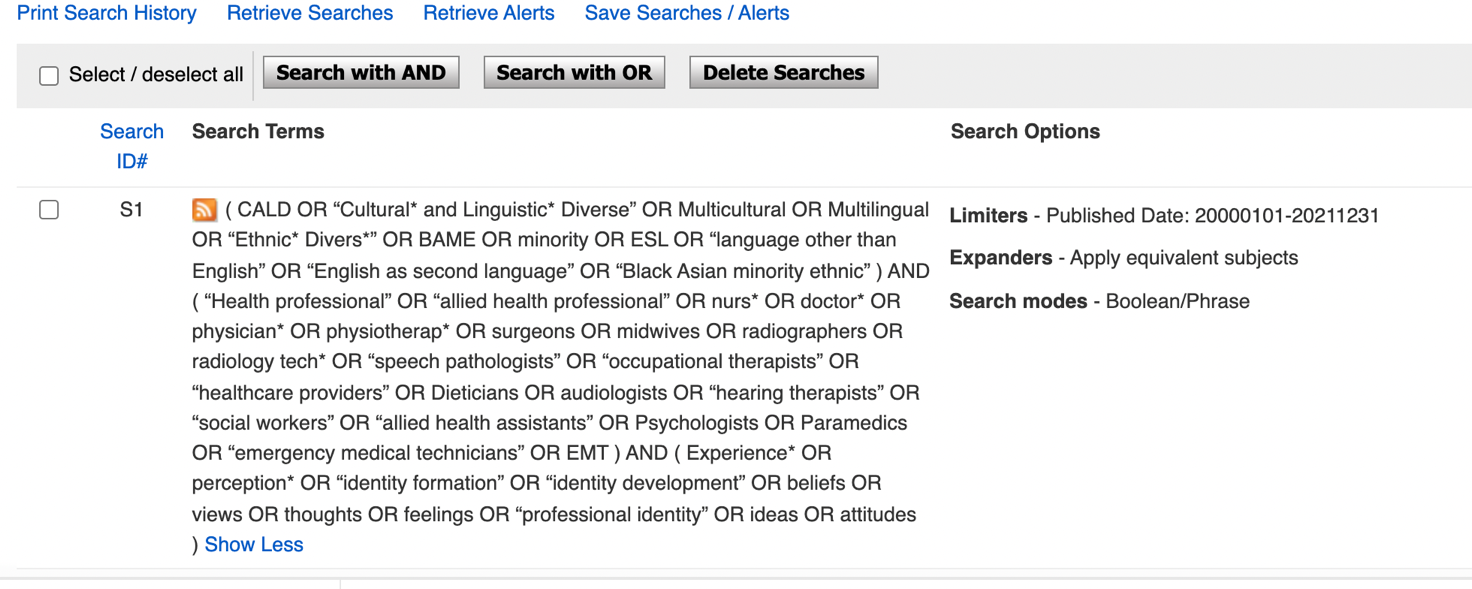
*
